# Current Strategies for Management of Medulloblastoma

**DOI:** 10.3390/diagnostics13162622

**Published:** 2023-08-08

**Authors:** Michael D. Prados

**Affiliations:** Charles B. Wilson Professor of Neurological Surgery and Professor of Pediatrics, University of California San Francisco, 1450 3rd Street, San Francisco, CA 94150, USA; michael.prados@ucsf.edu

**Keywords:** medulloblastoma, treatment, clinical trials

## Abstract

Medulloblastoma (MB) is the most common malignant central nervous system tumor of childhood, which includes multiple molecular subgroups (4) and subtypes (8 to 12), each with different outcomes and potential therapy options. Long-term survival remains poor for many of the subtypes, with high late mortality risks and poor health-related quality of life. Initial treatment strategies integrate molecular subgroup information with more standard clinical and phenotypic factors to risk stratify newly diagnosed patients. Clinical trials treating relapsed disease, often incurable, now include multiple new approaches in an attempt to improve progression-free and overall survival.

## 1. Introduction

Medulloblastomas comprise >90% of pediatric embryonal tumors with an incidence that ranges from 5 to 11 cases per 1 million individuals [1]. They arise within and constitute approximately 40% of all posterior fossa tumors. As with other embryonal tumors, they have the propensity to disseminate through the brain and spinal cord. The diagnosis is based on MRI imaging of the brain and spine and confirmed with surgical histology and molecular features (Table 1). Post-operative staging includes repeat cranial axis imaging and sampling of the cerebral spinal fluid for circulating tumor cells. There are now at least four molecular subgroups—Wingless-activated (WNT), Sonic Hedgehog-activated (SHH), and non-WNT/non-SHH (including Groups 3 and 4) [2]. Each has distinct molecular profiles and clinical outcomes, as currently defined in the 2021 WHO classification [3,4]. In addition to the subgroups, there are between 8 and 12 subtypes, primarily involving SHH, group 3, and group 4. DNA methylation is now considered the most accurate approach for accurate diagnosis. Standard of care treatment is based upon the extent of disease burden, age, and molecular/pathology classification [5]. In general, this includes maximal safe surgical resection, craniospinal irradiation (CSI), and cytotoxic chemotherapy, typically cyclophosphamide, lomustine, cisplatin, and vincristine combinations. Younger children are treated with intensive chemotherapy following surgery, with the goal to avoid radiotherapy with its attendant neurocognitive late effects [6]. Outcomes generally are based on prognostic factors (Table 2). Overall survival typically is 80% for average risk (total or near total resection and no metastatic disease) and 60% for high-risk disease (all others) [7,8,9]. Lower survivals globally from low-and middle-income countries are due to the limitation of resources [10,11]. These outcomes are not insurmountable, however, as reported, India has shown 5-year 62% survival for high-risk disease, similar to those reported in North America and Europe [12,13]. Relapsed medulloblastoma is often incurable [14,15,16,17,18]. Long-term survival continues to be problematic due to secondary neoplasms and other treatment-related chronic medical toxicities, with a reported 15-year mortality rate of over 20% [19,20]. Medulloblastoma can also arise in the setting of hereditary cancer predisposition syndromes. Germline mutations occur in approximately 5% of all patients diagnosed with medulloblastoma. Mutations have been identified in APC, BRCA2, PALB2, PTCH1, SUFU, and TP53 [21]. Testing for germline alterations is becoming more prevalent and has implications for long-term follow-up of both the patient and family members. Typical alterations are shown in Table 3. 

Thus, the intent of therapy is both overall survival benefits, as well as improved quality of life. 

## 2. Up-Front Clinical Trials

Clinical trials for newly diagnosed diseases incorporate molecular subgroups in addition to clinical and radiological risk factors. Highlights of some (but not all) recent studies include the following:

ACNS0331 was a trial for average-risk disease (NCT00085735): it randomized children to either standard dose (23.4 Gy) or reduced dose (18 Gy) CSI [7]. Adjuvant chemotherapy included cisplatin, lomustine, and vincristine alternating with cyclophosphamide and vincristine. Results were inferior with the reduced radiation dose treatment, except for WNT-activated subgroups. 

ACNS0332 was a trial for newly diagnosed high-risk medulloblastoma (NCT00392327): it assessed the addition of carboplatin concomitantly with radiation as well as the role of isotretinoin as a pro-apoptotic agent. While isotretinoin did not affect patient outcomes, the study did include a sub analysis of molecular subgroups for both interventions; this analysis demonstrated a survival advantage with the addition of carboplatin to radiation in patients with group 3 medulloblastoma [22]. 

ACNS0334 was a trial using high-dose chemotherapy in young children (generally considered a high-risk group with some exceptions), which included induction treatment with three cycles of intensive chemotherapy (cisplatin, cyclophosphamide, vincristine, etoposide) randomizing to the use of high-dose methotrexate or no methotrexate, followed by three cycles of high-dose carboplatin and thiotepa with stem cell rescue. Radiotherapy was given at the preference of the treating physician. Patients treated with methotrexate had a better 5-year EFS (68.2% versus 45.8%) [23].

Head Start III was another trial using intensive treatment for younger children, also using intensive chemotherapy, using a regimen like ACNS0334 including high-dose methotrexate for all children, followed by one cycle of high-dose thiotepa, carboplatin, and etoposide. Radiation was allowed for children older than six, or those without complete response. The best outcome was for those with nodular desmoplastic disease (5-year EFS of 89%). Those with classical or large cell anaplastic disease had worse outcomes (5-year EFS of 26% and 38% respectively). Of particular interest, those with metastatic desmoplastic disease also had an excellent outcome (5-year EFS of 82%), suggesting that traditional risk staging can be overcome by high-dose chemotherapy approaches [24].

Head Start IV trial (NCT02875314) is a prospective randomized study that treated younger children based on molecular subtype and response to induction chemotherapy to compare the efficacy of one versus three tandem cycles of myeloablative therapy. A subset of children with localized, average-risk SHH medulloblastoma were recently reported in abstract form. This subgroup was non-randomly assigned to receive three cycles of induction chemotherapy (vincristine, cisplatin, cyclophosphamide, etoposide, and high-dose methotrexate) followed by consolidation with a single cycle of myeloablative chemotherapy (thiotepa, carboplatin, etoposide) and stem cell rescue. Patients with less than a complete response after three induction cycles received two additional cycles prior to consolidation therapy. A total of 28 children with localized SHH medulloblastoma were enrolled in the trial with a median age of 2.1 years. The estimated 3-year event-free (EFS) and overall survival (OS) was 96% (CI: 89–100%) and 100%, respectively. The estimated 3-year EFS for SHH subtype 1 and 2 patients was 100% and 95%, respectively (*p* = 0.65). None of the M0 SHH medulloblastoma patients received irradiation [25].

SJMB03 trial (NCT00085202) was a risk-adaptive trial for both average and high-risk disease. Craniospinal radiation dose was reduced to 23.4 Gy in average-risk patients, and 36–39 Gy given for high-risk disease. Four cycles of adjuvant high-dose chemotherapy were given with stem cell support. Overall, three low-risk groups (WNT, low-risk SHH, and low-risk combined groups 3 and 4) and two very high-risk groups (high-risk SHH and high-risk combined groups 3 and 4) were defined. All patients with the WNT subgroup fared excellently (100% 5-year and overall survival). For patients with the SHH subgroup, metastatic disease, or LC/anaplastic histology, *MYCN* or *GLI2* amplification, or chromosome 17p loss were all significantly associated with poor outcome (25% PFS at 5 years). Otherwise, SHH subgroup patients fared very well (>75% PFS at 5-years). For high-risk patients, PFS at 5 years was 40.6% for group 3, and 68.1% for group 4. For combined subgroups 3 and 4, further risk assessment, using a joint clinical plus subtype-driven approach (based upon methylation subtype profiling using previously described eight subtypes) was used to identify three risk groups: low (patients with M_0_ and subtype VII), intermediate (patients with M_0_ and subtype I, II, IV, V, VI, and VIII), and high (patients with M_+_ disease or subtype III or *MYC* amplified) [8]. Prospective evaluation of these molecular subtype-specific cohorts is needed to validate these findings.

CTRI/2017/12/010767 (Clinical Trials Registry of India) was a trial in newly diagnosed, non-metastatic WNT tumors, attempting to omit brain and spine radiotherapy in this good-risk disease. Following surgery and staging, focal radiotherapy to the tumor bed only was given, followed by more standard adjuvant chemotherapy [26]. The study was closed early after seven patients were accrued, with three having a failure in the brain and/or spine. 

NCT02212574 was a trial that attempted radiotherapy avoidance by using chemotherapy only in the WNT subgroup disease but was halted prematurely due to an unacceptably high rate of relapse [27]. 

ACNS1422 (NCT02724579) and FOR-WNT2 (NCT04474964) are trials testing reduced-dose craniospinal irradiation (18 Gy) plus fewer maintenance cycles of adjuvant chemotherapy for low-risk WNT-driven tumors to further de-intensify treatment in this good-risk subgroup. ACNS1422 also omits the use of vincristine during radiotherapy. Results are pending. 

St. Jude SJMB12 clinical trial for newly diagnosed medulloblastoma (NCT01878617), as well as the International Society of Pediatrics Oncology (SIOP) medulloblastoma trials PNET 5 MB-LR and PNET 5 MB-SR, are ongoing risk-stratified protocols, assigning risk according to both clinical and molecular features. The results of those trials are eagerly awaited.

There have been many other trials for newly diagnosed disease, and in general, seem to confirm the use of the four major subgroups as stratification risks, to be combined with clinical features. All have shown excellent outcomes in WNT-driven disease, but even in this setting, radiotherapy seems required for long-term benefit. Within the other subgroups, further refinement of risk, now using methylation subgroup profiling (including subtypes as well) along with clinical features, is needed. 

## 3. Strategies at Relapse

Standard and more high-dose chemotherapy studies have been described in relapsed disease. Unfortunately, while some show efficacy in the short term, few appear curative, with a clear highly unmet need. 

One commonly used chemotherapy approach is based upon ACNS0821, a randomized phase 2 trial evaluating the use of temozolomide and irinotecan with or without bevacizumab in relapsed medulloblastoma. Median OS was 13 months in the standard arm and 19 months with the addition of bevacizumab; median event-free survival (EFS) was 6 months and 9 months, respectively [28]. 

Another chemotherapy approach uses an anti-angiogenic strategy in relapsed medulloblastoma (and other tumors) called MEMMAT, which evaluates the use of biweekly intravenous bevacizumab in combination with five oral drugs (thalidomide, celecoxib, fenofibrate, and alternating cycles of daily low-dose oral etoposide and cyclophosphamide), augmented with alternating courses of intrathecal etoposide and cytarabine (NCT01356290). This regimen, while seemingly complicated, is well tolerated and carried out as outpatient therapy. Recently, a MEMMAT “like” protocol result was just published in a group of children with relapsed medulloblastoma before the NCT01356290 trial was initiated. Treatment consisted of daily oral thalidomide, fenofibrate, celecoxib, and alternating 21-day cycles of low-dose oral etoposide and cyclophosphamide supplemented by IV bevacizumab and intraventricular therapy consisting of alternating etoposide and liposomal cytarabine. Median overall survival (OS) after recurrence for the whole group was 29.5 months with OS of 48.3 ± 9.3% at three years and progression-free survival of 42.0 ± 9.5% at three years [29]. 

Subgroup-specific small molecule targeted strategies are clearly needed in patients with relapsed disease. These studies are complicated by the small number of cases, heterogenous subtypes, and lack of available tumor tissue at the time of relapse. In the case of SHH diseases, multiple mutations exist, including *PTCH1*, suppressor of fused homologue (*SUFU*), Smoothened (*SMO*), and *GLI* in a high proportion of tumors. High-level amplifications may also be associated including *MYCL*, *GLI2*, *PPM1D*, *YAP1*, and *MDM4*, as well as MYCN expression. Several studies have evaluated the use of the SHH inhibitors vismodegib or sonidegib in patients with relapsed SHH-MB [30,31,32]. Unfortunately, sustained responses, when seen, are infrequent. As mentioned, intra-subgroup heterogeneity exists within SHH tumors, as with other subgroups. For instance, the response to SHH inhibitors is found specifically in patients with mutations upstream of Smoothened (SMO) [33]. Pre-clinical studies to define specificity (along with the required biomarkers) along with knowledge of resistance mechanisms are required in order to prioritize potential agents and to specifically stratify patients into targeted agent studies. Targeted agents can cause late effects, such as irreversible growth plate fusions, that also need to be considered when using these agents [34]. At this point, single-agent small molecule therapies do not appear sufficient to control disease at relapse.

The Pacific Pediatric Neuro-Oncology Consortium (PNOC) is evaluating a number of strategies for the management of relapsed medulloblastoma. A more patient-specific precision approach may be reasonable to consider, given that preclinical testing, including sophisticated molecular profiling, may not provide sufficient evidence to support single-agent targeted clinical testing [35]. PNOC027 (NCT05057702) is a pilot trial of real-time drug screening combined with genomic testing to determine an individualized treatment plan for these patients. Surgically acquired fresh tumor tissue removed at the time of relapse is prepared and sent to a CLIA-approved laboratory for drug testing, using a predefined medulloblastoma panel of drugs. A per-patient molecular tumor board reviews the results of the drug screen and sequencing, allowing up to four FDA-approved agents to be used in combination. The drugs chosen are primarily based on the potency of the assay (lowest IC_50_, and percent viability), potential for blood–brain barrier penetration, drug class, and toxicity. The intent is to recommend drugs that are highly potent and likely to reach therapeutic levels in tumors, along different classes, with minimal overlapping toxicity. While designed as a feasibility study, the trial will also assess safety as well as any preliminary efficacy signals using objective response as the readout. Enrollment has just begun, and results are anticipated by late 2024. 

The interplay of chromatin remodeling, *MYC* overexpression, and histone deacetylase (HDAC) inhibitors have suggested therapeutic strategies for MYC-driven medulloblastoma [36,37]. In addition, PI3K/AKT/mTOR signaling is also altered, with preclinical evidence that inhibitors can target medulloblastoma and decrease growth in vitro and in vivo, with synergistic effects combined with HDAC inhibitors [38,39,40]. Given these preclinical data, PNOC016 (NCT03893487) was designed as both a target validation study (the primary objective) and a preliminary efficacy trial to evaluate drug penetration of the pan-HDAC and PI3K inhibitor fimepinostat for newly diagnosed diffuse intrinsic pontine glioma, recurrent high-grade glioma, and recurrent medulloblastoma. Patients who are surgical candidates are eligible and receive the drug two days prior and on the day of surgery for the phase 0 PK/PD component of the trial and then continue the drug following surgical recovery. Accrual has now ended, with results expected soon. 

While cytotoxic therapies and molecularly targeted agents are important to explore, Immune modulation also continues to be investigated in medulloblastoma, including the use of viruses, CAR T cell-based strategies, vaccines, and other approaches. 

The measles virus is a negative strand, RNA virus, whose genome includes six protein products. Three of these proteins participate in the formation of the viral envelope; the H-protein is the surface glycoprotein that mediates measles virus attachment to its receptors, the CD46 molecule, and the SLAM receptor (the latter being predominantly present on activated B and T cells) and the recently identified nectin-4 receptor. During natural infection, the virus replicates in susceptible tissues causing a cytopathic effect, with the development of multinucleated giant cells (syncytia). Cells infected can cause fusion not only with other infected cells but also with uninfected neighboring cells [41,42,43]. Human studies have been carried out in adult glioma and other solid tumors, given intravenously, intratumorally, and intraperitoneally and found to be safe with some degree of single-agent efficacy. Based upon the preclinical and adult clinical trial data PNOC005 (NCT02962167) was designed as a phase 1 trial investigating the safety of modified measles virus (MV-NIS) in patients with recurrent medulloblastoma, given either directly into the tumor at the time of surgical resection, or via the CSF via lumbar injections. The initial cohorts of patients received a single infusion (CSF or tumor), while a second group was allowed up to two CSF infusions. The primary endpoint was safety. Preliminary data support the safety of these delivery approaches, suggesting that this oncolytic virus could potentially be combined with other agents/strategies. Patient accrual has been completed, and results are being compiled.

Chimeric antigen receptor (CAR) T cell strategies also continue to be investigated. Targets include HER2, B7-H3, EPHA2, GD2, PRAME207, and Interleukin 13 receptor *α*2, most of which are known to be expressed on medulloblastoma tumor cells. HER2 (NCT03500991) and B7-H3 (NCT04185038) CAR-T cell trials are ongoing [44,45,46]. EPHA2, HER2, and interleukin 13 receptors have all been identified as cell-surface targets expressed in medulloblastoma, with preclinical evidence to support a combination study [47,48]. While cost and patient access remain significant limitations, early experience using these CARs have been instrumental in optimizing CAR designs, management of toxicities, and CNS delivery strategies. Anecdotal reports of efficacy support continued clinical development in medulloblastoma and other CNS tumors. This is a fast-moving clinical effort, and larger sample size prospective trial results are eagerly anticipated.

PNOC025 (NCT05169944), is a recently opened phase 1 trial of the anti-CD47 monoclonal antibody Magrolimab, targeting part of the signal-regulatory protein (SIRP) *α*-CD47 pathway. CD47 is a phagocytosis checkpoint expressed on macrophages. Magrolimab blocks CD47 signaling and allows for macrophage-mediated phagocytosis in a number of tumor types, including medulloblastoma [49]. This trial just began enrollment, and while a safety study, it also incorporates unique imaging biomarkers to assess macrophage engagement in tumor regions. A potential future trial being considered will combine this antibody with other checkpoint inhibitors and/or cytotoxic agents, as a multi-targeted approach. As part of this and other PNOC clinical trials, as either secondary or exploratory endpoints, cell-free tumor DNA in both CSF and blood are collected longitudinally as an approach to assess tumor burden, to correlate both with imaging findings, and potentially early efficacy signals of treatment response [50,51].

PNOC028 is a soon-to-open phase 1 study evaluating the safety and efficacy of intra-tumoral injections of ex vivo expanded natural killer (NK) cells in children and young adults with recurrent supratentorial malignant brain tumors, including embryonal tumors. NK cells constitute between 5 and 15% of the peripheral blood lymphocyte population and have cytotoxic and regulatory activity [52]. They participate in cancer cell recognition through antibody-mediated cellular cytotoxicity (ADCC) and recognize infected cells or cancer cells that express danger signals including stress ligands, viral proteins, and antibodies. One main aspect that differentiates NK cells from T lymphocytes is that their recognition of targets does not depend on HLA antigen presentation. In this regard, they bypass the critical requirements for the therapeutic benefit of antigen-directed immunotherapies such as tumor-infiltrating lymphocytes, vaccines, and checkpoint inhibitors. Instead, they express receptors that allow for their recognition of malignant transformation but also control for self-tolerance. In addition to their direct anti-tumor activity, NK cells release interferon-γ and other cytokines, which result in several anti-cancer effects through crosstalk with the adaptive immune system, including activation of T-cells and dendritic cells, T-cell migration to the cancer and B-cell maturation. 

NK cells have also shown in vitro and in vivo activity against brain tumors, including GBM and medulloblastoma [53,54] and more importantly that injection to the contralateral side resulted in the migration of the NK cells across the normal brain into the tumor site. The group at MD Anderson Cancer Center (MDACC) initiated a Phase I clinical trial (NCT02271711) to expand and infuse autologous ex vivo expanded NK cells locoregionally into the fourth ventricle in children who have undergone resection of recurrent infratentorial tumors, including medulloblastoma. Results are pending for that trial.

A key contributor to intracranial tumor immunosuppression is transforming growth factor-beta (TFGβ) secreted by tumor cells and tumor-associated macrophages. The group at Nationwide described a modification to a previous method that enhances NK cell function and overcomes TGFβ-induced suppression (referred to as TGFβ “imprinting” (TGFβi)) by chronically stimulating the NK cells with TGFβ during the expansion process [55]. The resulting TGFβi NK cells exhibit high cytotoxicity and a pro-inflammatory hypersecretion of interferon-γ and tumor necrosis factor-alpha in response to tumor targets. In addition, this group has been able to identify blood donor pools with specific genotyping and HLA typing to allow for an “off the shelf” product, rather than relying upon autologous expansion of individual patient NK cells. Thus, PNOC028 will use this optimized NK cell product, evaluating the tolerability and feasibility of intra-tumoral infusions of expanded ex vivo TGFβi NK cells via an Ommaya reservoir in patients with recurrent or progressive supratentorial malignant brain tumors. Protocol activation is pending final FDA and IRB approvals.

These and many other ideas beyond the scope of this review have surfaced over the last decade, given the intense desire and clear need to define new therapeutic options for high-risk relapsed disease. A “one size fits all” approach is no longer relevant given the degree of biologic differences that exist with subgroups and clearly within individual patients. A major challenge going forward is to optimize the use of clinical trial designs that take into account both the rarity and heterogeneity found in relapsed disease.

## 4. Conclusions

Medulloblastoma is a complex, heterogeneous group of tumors with multiple subgroups and subtypes, each with important biologic and clinical differences. Much more preclinical data is needed to understand the underlining biology more fully, both at initial diagnosis and at the time of relapse. Relevant animal models need to incorporate these differences, in order to better rank and prioritize treatment. Ideally, combination approaches would be developed, targeting multiple pathways and targets, including cytotoxic (chemotherapy and radiotherapy), small molecule targeted therapy, and immunotherapy strategies. As therapies are being developed, efforts to comprehensively understand their effects in relation to this heterogeneity, as well as changes induced over time by treatment, are required to best understand their successes and failures. Clinical researchers can use this information to more specifically risk stratify and personalize treatment. For some tumors (e.g., WNT driven), the research is directed toward de-intensifying treatment; for others, better outcomes are needed and thus improved therapeutics. Late effects are critical to keep in mind, including secondary malignancies and other end-organ diseases, as well as neurocognitive sequelae [56].

## Figures and Tables

**Table 1:** Pathology.

*Medulloblastoma, molecularly defined*
Medulloblastoma, WNT-activated
Medulloblastoma, SHH-activated and TP53-wild type
Medulloblastoma, SHH-activated and TP53-mutant.
Medulloblastoma, non-WNT/non-SHH.

*Medulloblastoma, histologically defined*
Desmoplastic nodular medulloblastoma
Medulloblastoma with extensive nodularity
Large cell medulloblastoma
Anaplastic medulloblastoma

**Table 2:** Prognostic Factors.

Age at Diagnosis (high risk < 3 years of age; exception for desmoplastic medulloblastoma/medulloblastoma with extensive nodularity (MBEN))
Tumor histopathology (high risk: large cell/anaplastic variant)
Extent of CNS disease at diagnosis (high risk: >1.5 cm residual disease, and/or metastatic disease in brain and spine)
Biological/molecular tumor cell characteristics (high risk: group 3 and group 4 subgroups, SHH withTP53 alteration; lower risk: SHH with wild-type TP53 and WNT subgroup)

**Table 3:** Associated germ-line alterations.

Li-Fraumeni Syndrome (TP53 gene)
BRCA-associated tumors (BRCA2,PALB2 gene)
Turcot Syndrome (APC gene)
Gorlin Syndrome (PTCH1 and SUFU gene)
Rubinstein-Taybi Syndrome (CREBBP gene)

## References

[1] Ostrom Q.T., Cioffi G., Waite K., Kruchko C., Barnholtz-Sloan J.S. (2021). CBTRUS Statistical report: Primary brain and other central nervous system tumors diagnosed in the United States in 2014–2018. Neuro-Oncology.

[2] Taylor M.D., Northcott P.A., Korshunov A., Remke M., Cho Y.-J., Clifford S.C., Eberhart C.G., Parsons D.W., Rutkowski S., Gajjar A. (2012). Molecular subgroups of medulloblastoma: The current consensus. Acta Neuropathol..

[3] Louis D.N., Perry A., Reifenberger G., Von Deimling A., Figarella-Branger D., Cavenee W.K., Ohgaki H., Wiestler O.D., Kleihues P., Ellison D.W. (2016). The 2016 World health organization classification of tumors of the central nervous system: A summary. Acta Neuropathol..

[4] Louis D.N., Perry A., Wesseling P., Brat D.J., Cree I.A., Figarella-Branger D., Hawkins C., Ng H.K., Pfister S.M., Reifenberger G. (2021). The 2021 WHO classification of tumors of the central nervous system: A summary. Neuro-Oncology.

[5] Chang C.H., Housepian E.M., Herbert C. (1969). An operative staging system and a megavoltage radiotherapeutic technic for cerebellar medulloblastomas. Radiology.

[6] Rutkowski S., Cohen B., Finlay J., Luksch R., Ridola V., Valteau-Couanet D., Hara J., Garre M.L., Grill J. (2010). Medulloblastoma in young children. Pediatr. Blood Cancer.

[7] Michalski J.M., Janss A.J., Vezina L.G., Smith K.S., Billups C.A., Burger P.C., Embry L.M., Cullen P.L., Hardy K.K., Pomeroy S.L. (2021). Children’s oncology group phase III Trial of reduced-dose and reduced-volume radiotherapy with chemotherapy for newly diagnosed average-risk medulloblastoma. J. Clin. Oncol..

[8] Gajjar A., Robinson G.W., Smith K.S., Lin T., Merchant T.E., Chintagumpala M., Mahajan A., Su J., Bouffet E., Bartels U. (2021). Outcomes by clinical and molecular features in children with medulloblastoma treated with risk-adapted therapy: Results of an international phase III Trial (SJMB03). J. Clin. Oncol..

[9] Packer R.J., Sutton L.N., Goldwein J.W., Perilongo G., Bunin G., Cohen B.H., Kramer E.D., Zimmerman R.A., Rorke L.B., Evans A.E. (1991). Improved survival with the use of adjuvant chemotherapy in the treatment of medulloblastoma. J. Neurosurg..

[10] Rajagopal R., Abd-Ghafar S., Ganesan D., Mainudin A.Z.B., Wong K.T., Ramli N., Jawin V., Lum S.H., Yap T.Y., Bouffet E. (2017). Challenges of treating childhood medulloblastoma in a country with limited resources: 20 years of experience at a single tertiary center in Malaysia. J. Glob. Oncol..

[11] Mehrvar A., Tashvighi M., Asl A.A.H., Niktoreh-Mofrad N., Mehrvar N., Afsar N., Naderi A., Allebouyeh M., Qaddoumi I., Faranoush M. (2018). Management and outcomes of treating pediatric medulloblastoma: An eight years’ experience in an Iranian pediatric center. Child’s Nerv. Syst..

[12] Gupta T., Sinha S., Chinnaswamy G., Vora T., Prasad M., Bhat V., Goda J.S., Krishnatry R., Chatterjee A., Epari S. (2021). Safety and efficacy of concurrent carbo-platin during full-dose craniospinal irradiation for high-risk/metastatic medulloblastoma in a resource-limited setting. Pediatr. Blood Cancer.

[13] Kaur K., Kakkar A., Kumar A., Mallick S., Julka P.K., Gupta D., Suri A., Suri V., Sharma M.C., Sarkar C. (2016). Integrating molecular subclassification of medulloblastomas into routine clinical practice: A simplified approach. Brain Pathol..

[14] Chintagumpala M., Gajjar A. (2015). Brain tumors. Pediatr. Clin. N. Am..

[15] Pizer B., Donachie P.H., Robinson K., Taylor R.E., Michalski A., Punt J., Ellison D.W., Picton S. (2011). Treatment of recurrent central nervous system primitive neuroectodermal tumors in children and adolescents: Results of a Children’s Cancer and Leukaemia Group study. Eur. J. Cancer.

[16] Gururangan S., Krauser J., Watral M.A., Driscoll T., Larrier N., Reardon D.A., Rich J.N., Quinn J.A., Vredenburgh J.J., Desjardins A. (2008). Efficacy of high-dose chemotherapy or standard salvage therapy in patients with recurrent medulloblastoma. Neuro-Oncology.

[17] Dunkel I.J., Gardner S.L., Garvin J.H., Goldman S., Shi W., Finlay J.L. (2010). High-dose carboplatin, thiotepa, and etoposide with autologous stem cell rescue for patients with previously irradiated recurrent medulloblastoma. Neuro-Oncology.

[18] Koschmann C., Bloom K., Upadhyaya S., Geyer J.R., Leary S.E. (2016). Survival after relapse of medulloblastoma. J. Pediatr. Hematol..

[19] Salloum R., Chen Y., Yasui Y., Packer R., Leisenring W., Wells E., King A., Howell R., Gibson T.M., Krull K.R. (2019). Late morbidity and mortality among medulloblastoma survivors diagnosed across three decades: A report from the childhood cancer survivor study. J. Clin. Oncol..

[20] King A.A., Seidel K., Di C., Leisenring W.M., Perkins S.M., Krull K.R., Sklar C.A., Green D.M., Armstrong G.T., Zeltzer L.K. (2017). Long-term neurologic health and psychosocial function of adult survivors of childhood medulloblastoma/PNET: A report from the Childhood Cancer Survivor Study. Neuro-Oncology.

[21] Waszal S.M., Northcott P.A., Buchhalter I., Robinson G.W., Sutter C., Groebner S., Grund K.B., Brugières L., Jones D.T.W., Pajtler K.W. (2018). Spectrum and prevalence of genetic predisposition in medulloblastoma: A retrospective genetic study and prospective validation in a clinical trial cohort. Lancet Oncol..

[22] Leary S.E.S., Packer R.J., Li Y., Billups C.A., Smith K.S., Jaju A., Heier L., Burger P., Walsh K., Han Y. (2021). Efficacy of carboplatin and isotretinoin in children with high-risk medulloblastoma: A randomized clinical trial from the children’s oncology group. JAMA Oncol..

[23] Mazewski C., Kang G., Kellie S., Gossett J., Leary S., Li B., Aridgides P., Hayes L., Reddy A., Shaw D. (2020). Efficacy of methotrexate (MTX) according to molecular sub-type in young children with medulloblastoma (MB): A report from children’s oncology group phase III trial ACNS0334. Neuro-Oncology.

[24] Dhall G., O’Neil S.H., Ji L., Haley K., Whitaker A.M., Nelson M.D., Gilles F., Gardner S.L., Allen J.C., Cornelius A.S. (2020). Excellent outcome of young children with nodular desmoplastic medulloblastoma treated on “Head Start” III: A multi-institutional, prospective clinical trial. Neuro-Oncology.

[25] Dhall G., Stanek J., Blue M., Patel P., Thomas D., Pierson C., Tamrazi B., Mahadeo K.M., Fleming J., Bell E. (2022). LTBK-05. Outcomes of infants and young children with newly diagnosed localized (M0) SHH Medulloblastoma Treated on The NEXT Consortium “Head Start” 4 Protocol. Neuro-Oncology.

[26] Gupta T., Pervez S., Dasgupta A., Chatterjee A., Epari S., Chinnaswamy G., Jalali R. (2022). Omission of upfront craniospinal irradiation in patients with low-risk WNT-pathway medulloblastoma is associated with unacceptably high risk of neuraxial failure. Clin. Cancer Res..

[27] Cohen K., Bandopadhayay P., Chi S., London W., Rodriguez F., Hawkins C., Yang E., Aguilera D., Castellino R., MacDonald T. (2019). Pilot study of a surgery and chemotherapy-only approach in the upfront therapy of children with WNT-positive standard risk medulloblastoma. Neuro-Oncology.

[28] Levy A.S., Krailo M., Chi S., Villaluna D., Springer L., Williams-Hughes C., Fouladi M., Gajjar A. (2021). Temozolomide with irinotecan versus temozolomide, irinotecan plus bevacizumab for recurrent medulloblastoma of childhood: Report of a COG randomized Phase II screening trial. Pediatr. Blood Cancer.

[29] Slavc I., Mayr L., Stepien N., Gojo J., Aliotti Lippolis M., Azizi A.A., Chocholous M., Baumgartner A., Hedrich C.S., Holm S. (2021). Improved long term survival with recurrent medulloblastoma treated with a “MEMMAT-like” metronomic antiangiogenic approach. Cancers.

[30] Robinson G.W., Orr B.A., Wu G., Gururangan S., Lin T., Qaddoumi I., Packer R.J., Goldman S., Prados M.D., Desjardins A. (2015). Vismodegib exerts targeted efficacy against recurrent sonic hedgehog-subgroup medulloblastoma: Results from phase II pediatric brain tumor consortium studies PBTC-025B and PBTC-032. J. Clin. Oncol..

[31] Gajjar A., Stewart C.F., Ellison D.W., Kaste S., Kun L.E., Packer R.J., Goldman S., Chintagumpala M., Wallace D., Takebe N. (2013). Phase I study of vismodegib in children with recurrent or refractory medulloblastoma: A pediatric brain tumor consortium study. Clin. Cancer Res..

[32] Kieran M.W., Chisholm J., Casanova M., Brandes A.A., Aerts I., Bouffet E., Bailey S., Leary S., MacDonald T.J., Mechinaud F. (2017). Phase I study of oral sonidegib (LDE225) in pediatric brain and solid tumors and a phase II study in children and adults with relapsed medulloblastoma. Neuro-Oncology.

[33] Kool M., Jones D.T., Jager N., Northcott P.A., Pugh T.J., Hovestadt V., Piro R.M., Esparza L.A., Markant S.L., Remke M. (2014). Genome sequencing of SHH medulloblastoma predicts genotype-related response to smoothened inhibition. Cancer Cell.

[34] Robinson G.W., Kaste S.C., Chemaitilly W., Bowers D.C., Laughton S., Smith A., Gottardo N.G., Partap S., Bendel A., Wright K.D. (2017). Irreversible growth plate fusions in children with medulloblastoma treated with a targeted hedgehog pathway inhibitor. Oncotarget.

[35] Cavalli F.M.G., Remke M., Rampasek L., Peacock J., Shih D.J., Luu B., Garzia L., Torchia J., Nor C., Morrissy A.S. (2017). Intertumoral heterogeneity within medulloblastoma subgroups. Cancer Cell.

[36] Dubuc A.M., Remke M., Korshunov A., Northcott P.A., Zhan S.H., Mendez-Lago M., Kool M., Jones D.T.W., Unterberger A., Morrissy A.S. (2013). Aberrant patterns of H3K4 and H3K27 histone lysine methylation occur across subgroups in medulloblastoma. Acta Neuropathol..

[37] Northcott P.A., Nakahara Y., Wu X., Feuk L., Ellison D.W., Croul S., Mack S., Kongkham P.N., Peacock J., Dubuc A. (2009). Multiple recurrent genetic events converge on control of histone lysine methylation in medulloblastoma. Nat. Genet..

[38] Pei Y., Liu K.W., Wang J., Garancher A., Tao R., Esparza L.A., Maier D.L., Udaka Y.T., Murad N., Morrissy S. (2016). HDAC and PI3K Antagonists Cooperate to Inhibit Growth of MYC-Driven Medulloblastoma. Cancer Cell.

[39] Ehrhardt M., Craveiro R.B., Holst M.I., Pietsch T., Dilloo D. (2015). The PI3K inhibitor GDC-0941 displays promising in vitro and in vivo efficacy for targeted medulloblastoma therapy. Oncotarget.

[40] Singh A.R., Joshi S., Zulcic M., Alcaraz M., Garlich J.R., Morales G.A., Cho Y.J., Bao L., Levy M.L., Newbury R. (2016). PI-3K Inhibitors Preferentially Target CD15+ Cancer Stem Cell Population in SHH Driven Medulloblastoma. PLoS ONE.

[41] Lal S., Carrera D., Phillips J.J., Weiss W.A., Raffel C. (2018). An oncolytic measles virus-sensitive Group 3 medulloblastoma model in immune-competent mice. Neuro-Oncology.

[42] Studebaker A.W., Hutzen B., Pierson C.R., Russell S.J., Galanis E., Raffel C. (2012). Oncolytic measles virus prolongs survival in a murine model of cerebral spinal fluid disseminated medulloblastoma. Neuro-Oncology.

[43] Studebaker A.W., Kreofsky C.R., Pierson C.R., Russell S.J., Galanis E., Raffel C. (2010). Treatment of medulloblastoma with a modified measles virus. Neuro-Oncology.

[44] Vitanza N.A., Johnson A.J., Wilson A.L., Brown C., Yokoyama J.K., Künkele A., Chang C.A., Rawlings-Rhea S., Huang W., Seidel K. (2021). Locoregional infusion of HER2-specific CAR T cells in children and young adults with recurrent or refractory CNS tumors: An interim analysis. Nat. Med..

[45] Majzner R.G., Theruvath J.L., Nellan A., Heitzeneder S., Cui Y., Mount C.W., Rietberg S.P., Linde M.H., Xu P., Rota C. (2019). CAR T Cells Targeting B7-H3, a pan–cancer antigen, demonstrate potent preclinical activity against pediatric solid tumors and brain tumors. Clin. Cancer Res..

[46] Donovan L.K., Delaidelli A., Joseph S.K., Bielamowicz K., Fousek K., Holgado B.L., Manno A., Srikanthan D., Gad A.Z., Van Ommeren R. (2020). Locoregional delivery of CAR T cells to the cerebrospinal fluid for treatment of metastatic medulloblastoma and ependymoma. Nat. Med..

[47] Flores C., Wildes T., Dean B.D., Moore G., Drake J., Abraham R., Gil J., Yegorov O., Yang C., Dean J. (2019). Massive clonal expansion of medulloblastoma-specific T cells during adoptive cellular therapy. Sci. Adv..

[48] Pham C.D., Flores C., Yang C., Pinheiro E.M., Yearley J.H., Sayour E.J., Pei Y., Moore C., McLendon R.E., Huang J. (2016). Differential immune microenvironments and response to immune checkpoint blockade among molecular subtypes of murine medulloblastoma. Clin. Cancer Res..

[49] Gholamin S., Mitra S.S., Feroze A.H., Liu J., Kahn S.A., Zhang M., Esparza R., Richard C., Ramaswamy V., Remke M. (2017). Disrupting the CD47-SIRPalpha anti-phagocytic axis by a humanized anti-CD47 antibody is an efficacious treatment for malignant pediatric brain tumors. Sci. Transl. Med..

[50] Liu A.P.Y., Smith K.S., Kumar R., Paul L., Bihannic L., Lin T., Maass K.K., Pajtler K.W., Chintagumpala M., Su J.M. (2021). Serial assessment of measurable residual disease in medulloblastoma liquid biopsies. Cancer Cell.

[51] Sun Y., Li M., Ren S., Liu Y., Zhang J., Li S., Gao W., Gong X., Liu J., Wang Y. (2021). Exploring genetic alterations in circulating tumor DNA from cerebrospinal fluid of pediatric medulloblastoma. Sci. Rep..

[52] Kannan G.S., Aquino-Lopez A., Lee D.A. (2017). Natural killer cells in malignant hematology: A primer for the non-immunologist. Blood Rev..

[53] Castriconi R., Dondero A., Negri F., Bellora F., Nozza P., Carnemolla B., Raso A., Moretta L., Moretta A., Bottino C. (2007). Both CD133^+^ and CD133^−^ medulloblastoma cell lines express ligands for triggering NK receptors and are susceptible to NK-mediated cytotoxicity. Eur. J. Immunol..

[54] Fernández L., Portugal R., Valentín J., Martín R., Maxwell H., González-Vicent M., Díaz M.A., de Prada I., Pérez-Martínez A. (2013). In vitro natural killer cell immunotherapy for medulloblastoma. Front. Oncol..

[55] Foltz J.A., Moseman J.E., Thakkar A., Chakravarti N., Lee D.A. (2018). TGFβ Imprinting During Activation Promotes Natural Killer Cell Cytokine Hypersecretion. Cancers.

[56] Mertens A.C., Yasui Y., Neglia J.P., Potter J.D., Nesbit M.E., Ruccione K., Smithson W.A., Robison L.L. (2001). Late mortality experience in five-year survivors of childhood and adolescent cancer: The Childhood Cancer Survivor Study. J. Clin. Oncol..

